# Binding to The Target Cell Surface Is The Crucial Step in Pore Formation of Hemolysin BL from *Bacillus cereus*

**DOI:** 10.3390/toxins11050281

**Published:** 2019-05-20

**Authors:** Nadja Jessberger, Richard Dietrich, Stefanie Schwemmer, Franziska Tausch, Valerie Schwenk, Andrea Didier, Erwin Märtlbauer

**Affiliations:** Department of Veterinary Sciences, Faculty of Veterinary Medicine, Ludwig-Maximilians-Universität München, Schönleutnerstr. 8, 85764 Oberschleißheim, Germany; r.dietrich@mh.vetmed.uni-muenchen.de (R.D.); Stefanie.Schwemmer@mh.vetmed.uni-muenchen.de (S.S.); Franziska.Tausch@gmx.de (F.T.); Valerie.Schwenk@mh.vetmed.uni-muenchen.de (V.S.); A.Didier@mh.vetmed.uni-muenchen.de (A.D.); e.maertlbauer@mh.vetmed.uni-muenchen.de (E.M.)

**Keywords:** *Bacillus cereus*, enterotoxins, hemolysin BL, three-component toxin, mode of action

## Abstract

A major virulence factor involved in *Bacillus cereus* food poisoning is the three-component enterotoxin hemolysin BL. It consists of the binding component B and the two lytic components L_1_ and L_2_. Studying its mode of action has been challenging, as natural culture supernatants additionally contain Nhe, the second three-component enterotoxin, and purification of recombinant (r) Hbl components has been difficult. In this study, we report on pore-forming, cytotoxic, cell binding and hemolytic activity of recently generated rHbl components expressed in *E. coli*. It is known that all three Hbl components are necessary for cytotoxicity and pore formation. Here we show that an excess of rHbl B enhances, while an excess of rHbl L_1_ hinders, the velocity of pore formation. Most rapid pore formation was observed with ratios L_2_:L_1_:B = 1:1:10 and 10:1:10. It was further verified that Hbl activity is due to sequential binding of the components B - L_1_ - L_2_. Accordingly, all bioassays proved that binding of Hbl B to the cell surface is the crucial step for pore formation and cytotoxic activity. Binding of Hbl B took place within minutes, while apposition of the following L_1_ and L_2_ occurred immediately. Further on, applying toxin components simultaneously, it seemed that Hbl L_1_ enhanced binding of B to the target cell surface. Overall, these data contribute significantly to the elucidation of the mode of action of Hbl, and suggest that its mechanism of pore formation differs substantially from that of Nhe, although both enterotoxin complexes are sequentially highly related.

## 1. Introduction

*Bacillus cereus* is considered an emerging pathogen relevant for food poisoning worldwide [1,2,3]. While the emetic toxin cereulide causes vomiting, enterotoxins are responsible for the diarrheal form [4]. The enterotoxin complexes Nhe (non-haemolytic enterotoxin [5]) and Hbl (haemolysin BL [6]) are composed of three individual components each, which is a unique feature of the *Bacillus cereus* group [4,7]. The genes encoding Nhe can be detected in nearly all enteropathogenic *B. cereus* strains, the *hbl* genes in about 45–65%. Strains bearing only *hbl* have not yet been found [8,9,10]. 

The *hbl* genes are organized in the operon *hblCDA*, encoding the components Hbl L_2_, L_1_ and B, respectively [11]. Expression of the *hbl* operon is influenced by a variety of regulatory proteins, such as the major virulence regulator PlcR [12,13], CcpA [14], ResD [15] and Fnr [16,17]. When the Hbl components L_2_, L_1_ and B were first isolated from strain F837/76, they had a molecular weight of 45, 36 and 35 kDa, respectively [18]. Later it was shown that other *B. cereus* strains produce Hbl components of various sizes, and that in one strain more than one variant of each component can exist [19]. Two distinct homologs of Hbl L_2_, L_1_ and B were found in one *B. cereus* strain, leading to the assumption that two distinct homologs of the *hbl* operon exist [20]. The more prevalent variant additionally contains *hblB*, which is located downstream of *hblCDA* [11]. Since then, *hblB* was considered a pseudogene, as no transcription could be detected [12,21]. Only in 2010, it was shown by Clair and coworkers that the corresponding protein Hbl B’ is indeed secreted by the *B. cereus* strain ATCC 14579 [22]. The second, rarer variant of the *hbl* operon, named *hbl_a_*, contains only *hblCDA* [20].

The mode of action of Hbl is still not clear. The toxin is hemolytic, cytotoxic, dermonecrotic and active in vascular permeability tests [6,18]. Furthermore, it causes fluid accumulation in ligated rabbit ileal loops, and was therefore referred to as tripartite *B. cereus* diarrheal enterotoxin [6]. A recent study showed that Hbl induces inflammasome-mediated mortality in macrophages [23]. Pore formation was first shown by osmotic protection assays [24]. The same study suggested that all three Hbl components can bind individually to erythrocytes and form a “membrane attack complex”, which finally leads to cell lysis. A more recent study showed that only Hbl B can bind to the surface of Chinese hamster ovary (CHO) cells, and that only sequential binding in the order B - L_1_ - L_2_ leads to cytotoxic activity [25]. Solving the crystal structure of Hbl B, Madegowda and coworkers suggested that Hbl B alone might be able to bind to the cell surface, oligomerize and form a pore, just like the structurally very similar hemolysin E from *E. coli* [26,27,28]. The two components L_2_ and L_1_, which are also necessary to induce toxicity, were suggested to either stabilize Hbl B, induce conformational changes to B, or even enter the cell [26].

Another characteristic feature of Hbl is the discontinuous hemolysis zone phenomenon on blood agar [6,18,24]. When Hbl diffuses from a certain point in blood agar, lysis is first observed at a distance away from that point. This was explained by different diffusion velocities of the Hbl components, creating a continuous concentration gradient. Lysis starts at the point of the appropriate concentration ratio. Moreover, excess of Hbl L_1_ or B was shown to inhibit hemolytic activity [20,24]. All these studies were performed with Hbl components purified from *B. cereus* supernatant via anion exchange chromatography. With this approach, the purity of the single components could not be completely guaranteed. To exclude trace contaminations as well as biased results, the use of recombinant Hbl components was recommended [6,18,29]. Just recently we were able to overexpress and purify fully functional recombinant Hbl components (rHbl) in *E. coli*, which is highly advantageous, regarding more detailed studies on the mode of action of Hbl. Moreover, for the first time we demonstrated complex formation between Hbl L_1_ and B, as well as L_1_ and L_2_ in solution, prior to cell binding [30]. In the present study, the binding order and manners of the Hbl components were verified, the optimum concentration ratio for pore formation and toxic activity was determined, and the action of rHbl on sheep blood agar was investigated. Our data corroborate former suggestions that it is the B component of Hbl, particularly its binding ability to the cell surface, which substantially determines toxic activity.

## 2. Results

### 2.1. All Three Hbl Components Are Necessary for Cytotoxicity and Pore Formation

It is controversially discussed in literature whether or not two Hbl components alone might be able to exhibit toxic activity, or form a pore, as has been shown for the structurally-related Nhe components B and C [31]. In early spectrophotometric hemolysis assays, 37% hemolysis was detected with only Hbl B and L_2_; and vascular permeability tests showed minor edema with only Hbl B and L_1_ [18]. Other studies didn’t detect any activity with only two Hbl components [25]. 

**Figure 1:**
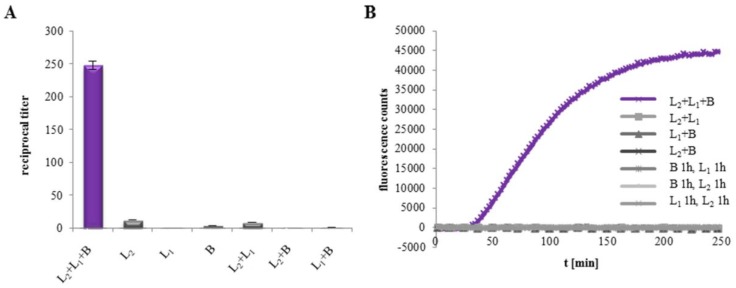
Toxic activity of the rHbl components on Vero cells. (**A**) WST-1 bioassay. The rHbl components were mixed in 1:1:1 ratios and applied as serial dilution to the cells, starting with 75 pmol/mL. Shown are reciprocal titers, which were defined as the dilution necessary to gain 50% dead cells after 24 h. Titers below 20 indicate no specific toxic activity. (**B**) PI influx test. The rHbl components were mixed in 1:1:1 ratios and applied to the cells (37.5 pmol/mL each). Alternatively, cells were incubated for 1 h with one component, washed and then incubated for another h with the second component. Only for the sample with rHbl L_2_+L_1_+B, PI influx and thus, pore formation was measured.

Figure 1 shows our results of WST-1 bioassays and propidium iodide (PI) influx tests on Vero cells with two rHbl components each. When combinations of rHbl L_1_ and B, L_2_ and B, or L_2_ and L_1_ were applied to the cells, neither toxic activity nor pore formation could be observed. We gained the same results when PI influx tests were performed under consecutive conditions, i.e., for 1 h each (Figure 1B). These data strongly indicate that any combination of only two of the three Hbl components is not able to induce pore formation, and thus, no toxic activity can be achieved on the tested target cells.

### 2.2. Velocity of Pore Formation Depends on rHbl Concentration

**Figure 2:**
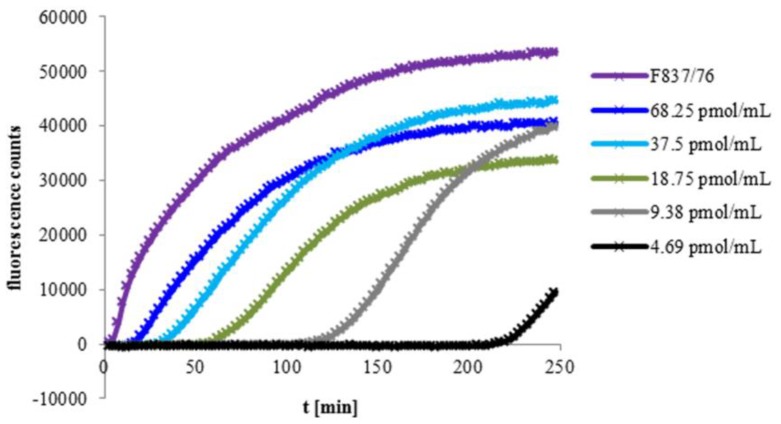
PI influx into Vero cells. The rHbl components were adjusted to the indicated molar concentrations, mixed in 1:1:1 ratios and applied to Vero cells. Increasing fluorescence was measured representing influx of propidium iodide. Supernatant of strain F837/76 was used as a control.

To determine the influence of the amount of Hbl on pore formation, rHbl components were mixed in 1:1:1 ratios and applied in specific molar concentrations (68.25, 37.5, 18.75, 9.38 and 4.69 pmol/mL each) to Vero cells. Increasing fluorescence was measured representing influx of propidium iodide (Figure 2). Supernatant of *B. cereus* strain F837/76, which causes rapid PI influx, was used as control. It was shown that the start point of PI influx and thus, pore formation, depends significantly on the Hbl concentration.

### 2.3. Excess of rHbl B Accelerates, Excess of rHbl L_1_ Hinders Pore Formation

**Figure 3:**
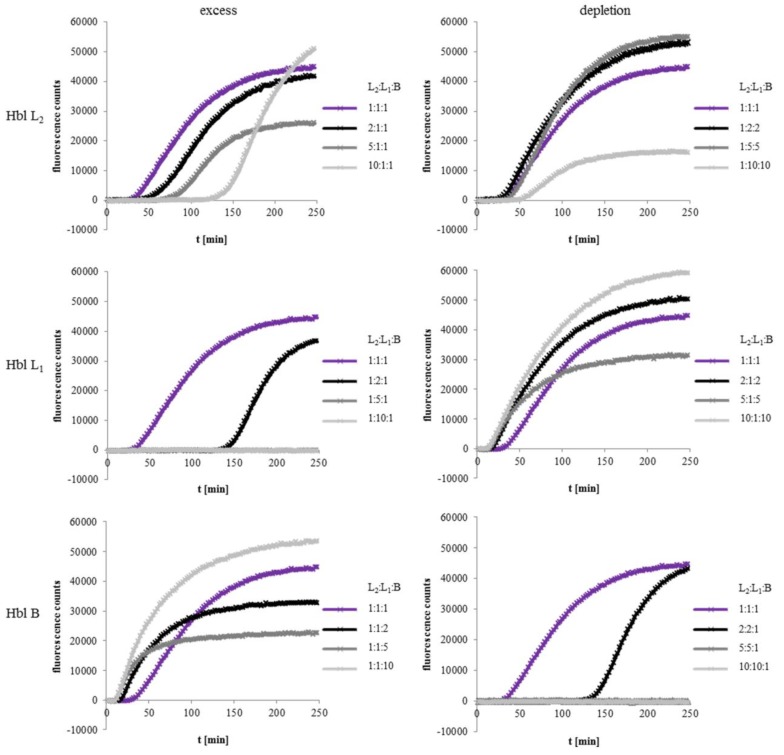
PI influx test on Vero cells with rHbl components L_2_, L_1_ and B mixed in different ratios. The components were pre-mixed in the ratio 1:1:1 or with 2×, 5× and 10× excess or depletion of each component compared to the other two. The 1:1:1 ratio corresponds to 37.5 pmol/mL each. Upper row: Results for Hbl L_2_. Middle row: Results for Hbl L_1_, lower row: Results for Hbl B. Left: 2× (black), 5× (dark grey) and 10× (light grey) excess of each Hbl component. Right: 2× (black), 5× (dark grey) and 10× (light grey) depletion of each Hbl component. The 1:1:1 (purple) sample is shown in every diagram as control.

To determine the ratio of rHbl L_2_:L_1_:B required for maximum activity, PI influx tests on Vero cells were carried out. The three components were pre-mixed in the ratio 1:1:1 (Figure 3, shown in purple as control in every diagram) or with 2×, 5× and 10× excess (Figure 3 left) or depletion (Figure 3 right) of each single component. Excess of rHbl L_2_ led to a delayed PI influx, while depletion of rHbl L_2_ up to factor 5 seemed not to influence the speed of pore formation (Figure 3 upper row). On the other hand, a 2× excess of rHbl L_1_ delayed PI influx significantly, at 5× and 10× excess, even no pore formation was detectable within 4 h. Depletion of L_1_ accelerated pore formation compared to the 1:1:1 control (Figure 3 middle row). With regards to rHbl B, pore formation started earlier when this component was applied in excess, while 2× depletion of B delayed PI influx significantly (Figure 3 lower row). At ratios of 5:5:1 or 10:10:1 no more pore formation was detectable within 4 h. Altogether, pore formation by Hbl seems to occur faster the more Hbl B is present compared to L_1_ and L_2_. On the contrary, excess of Hbl L_1_ seems to hinder pore formation. Reducing the amount of Hbl L_2_ seems not to be crucial, but high excess of that component also retards pore formation.

### 2.4. Hbl Activity Is Due to Sequential Binding of The Components B - L_1_ - L_2_ as Well as Their Concerted Action

**Figure 4:**
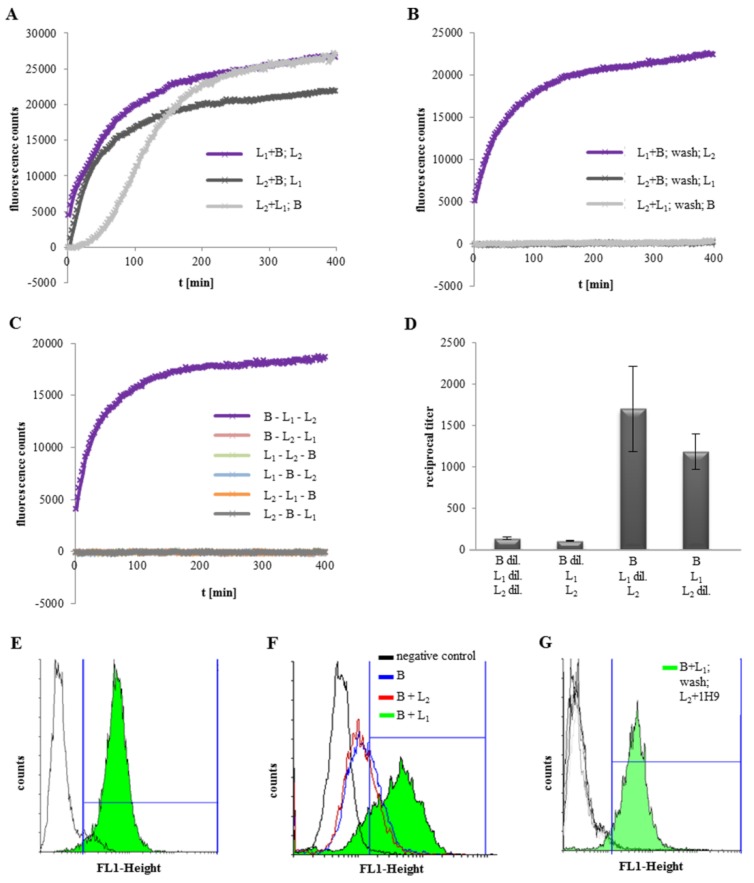
Toxicity of rHbl to Vero cells. If not stated otherwise, the rHbl components were used in molar concentrations of 37.5 pmol/mL each. (**A**) Two rHbl components were mixed in 1:1 ratio and applied to the cells. After 1 h, the corresponding third component was added. Immediately after that, PI influx was measured. (**B**) Two rHbl components were again mixed in a 1:1 ratio and applied to the cells. After 1 h, the mixture was removed and Vero cells were washed three times with medium. Subsequently the corresponding third component was added and the PI influx was measured. (**C**) Each component was added individually to the cells. After 1 h each, cells were washed three times in medium. After adding the third component, PI influx was measured immediately. (**D**) WST-1 bioassays on Vero cells. The single rHbl components were added consecutively to the cells with two intermediate washing steps with medium each time. Because of the high toxic activity in this approach, rHbl components were used in concentrations of 3.75 pmol/mL. In each setup, two components were applied constantly, and the third as a dilution series of 1:2. Each component was incubated for only 45 min. After the third component was applied, cells were again washed two times and incubated with WST-1 for 1.5 h before measuring. Titers were determined as the toxin dilution causing 50% dead cells (dil. = dilution series.). (**E**) Flow cytometry results of rHbl B binding to Vero cells. Cells were incubated successively for 1 h with rHbl B, mAb 1G8, and for 45 min with Alexa Fluor^®^ 488 goat anti mouse IgG. A fluorescence shift (green) was visible compared to the negative control (no rHbl B). (**F**) Flow cytometry results of rHbl B (blue), rHbl B+L_2_ (red) and rHbl B+L_1_ (green) detected with mAb 1B8 and Alexa Fluor^®^ 488 goat anti mouse IgG. (**G**) Flow cytometry results. Vero cells were incubated with 1. mAb 1H9, 2. rHbl L_2_+1H9, 3. rHbl B and L_2_+1H9, 4. rHbl L_1_ and L_2_+1H9 and 5. rHbl B+L_1_ and after washing L_2_+1H9. Only the latter showed fluorescence. Fluorescence was again detected with Alexa Fluor^®^ 488 goat anti mouse IgG.

The binding mechanism of Hbl to the target cell surface is still not clear. Individual binding of each component to erythrocytes has been suggested [24], as well as sequential binding to Chinese hamster ovary cells in the order B - L_1_ - L_2_ [25]. To investigate this, PI influx tests on Vero cells were set up in the first place. Initially, Vero cells were incubated for 1 h with two rHbl components and after that, the third component was added. PI influx, and thus pore formation, started earlier, the earlier rHbl B was added. Exchange of L_2_ and L_1_ as the last component had only minor influence (Figure 4A). In the next setup, Vero cells were washed three times in medium before addition of the third component. In this sequential approach, pore formation was only observed when rHbl B and L_1_ were added before, and L_2_ after washing (Figure 4B). In the final experiment the single components were added individually, with three washing steps in between. It became obvious that the Hbl components indeed assemble sequentially, and that the binding order B - L_1_ - L_2_ is obligatory for pore formation (Figure 4C). 

In WST-1 bioassays rHbl was also only toxic when applied in the order B - L_1_ - L_2_ (see Figure S1A). To assess the significance of the individual components for the toxic activity of Hbl, rHbl L_2_, L_1_ and B were either applied in a concentration of 3.75 pmol/mL per well, or as a dilution series of 1:2, starting with 3.75 pmol/mL. Each component was incubated for 45 min with the cells before washing and the addition of the next component. After the third component, cells were again washed two times and incubated with WST-1 for 1.5 h before measuring. Application of all three components individually as a dilution series resulted in a low reciprocal titer (136) compared to the dilution series of pre-mixed L_2_, L_1_ and B (titer 1:248; see Figure 1). When only rHbl B was diluted, the titer was 107. Dilution of rHbl L_1_ resulted in a titer of 1700, dilution of L_2_ in a titer of 1183 (Figure 4D). These data suggest that binding of Hbl B to the target cell surface is the crucial step for Hbl pore formation. High amounts of L_2_ or L_1_ do not advance pore formation and toxic activity, if there is not enough B bound to the cells. On the contrary, dilution of L_2_ and especially L_1_ even increase toxic activity, which supports the data from PI influx tests in Figure 3.

To further corroborate the conclusions about the sequential binding order, flow cytometry analyses were carried out. First, Vero cells were incubated successively with rHbl B, the correspondent mAb 1G8 and Alexa Fluor^®^ 488 goat anti mouse IgG. A significant increase of fluorescence was visible, compared to the negative control (Figure 4E and Table 1), indicating that rHbl B specifically binds to the cell surface. In a subsequent approach, rHbl B and L_1_ or L_2_ were mixed 1:1 and applied to the cells. For detection, mAb 1B8 and Alexa Fluor^®^ 488 goat anti mouse IgG were used. The mAb 1G8 (see above), which is best suited for detecting Hbl B on cell surfaces, was replaced, as it cross-reacts with L_1_ [30]. Interestingly, the number of fluorescence-positive cells was increased when rHbl B was chaperoned by L_1_ (Figure 4F and Table 1). To demonstrate the impact of rHbl L_2_, the neutralizing mAb 1H9 (anti L_2_) was used, together with different combinations of the rHbl components. Fluorescence was barely detected when Vero cells were incubated with only mAb 1H9 (negative control), rHbl L_2_+1H9, rHbl B and L_2_+1H9 or rHbl L_1_ and L_2_+1H9 (Figure 4G and Table 1). However, a strong increase of fluorescence was observed when the cells were first incubated with rHbl B+L_1_ and subsequently with rHbl L_2_+1H9 (Figure 4G and Table 1).

Repeating these experiments, PI was added to the cells. Only in the samples where mAb 1H9, L_2_, L_1_ and B were used, approximately 71% of the Vero cells were PI positive (see Figure S1B). Nevertheless, cells were morphologically still intact, firstly indicating that mAb 1H9 does not hinder assembly of the Hbl pore and PI influx, but presumably a quick lysis of the cells. Secondly, this approach confirmed again that Hbl B alone is not able to form pores. 

Altogether, these data show that rHbl B binds specifically to Vero cells and that the presence of rHbl L_1_ enhances this binding. rHbl L_2_ can only be detected at the cell surface when B and L_1_ are already present.

### 2.5. Binding of Hbl B to The Cell Surface Is The Crucial Step for Pore Formation

To determine the time necessary for each Hbl component to bind to the target cells, further PI influx tests were performed. The rHbl components were successively applied to the cells in concentrations of 37.5 pmol/mL each. Application order was B - L_1_ - L_2_. After adding the B and L_1_ components, the cells were washed twice in medium. After addition of L_2_, measurement was immediately started. When B as well as L_1_ were applied for 1 h, 30 or 15 min each, rapid PI influx was detected (data not shown). Shorter incubation times were first tested for the binding component B, with rHbl L_1_ being constantly applied for 10 min. Full PI influx was observed for 10 min incubation. 

**Figure 5:**
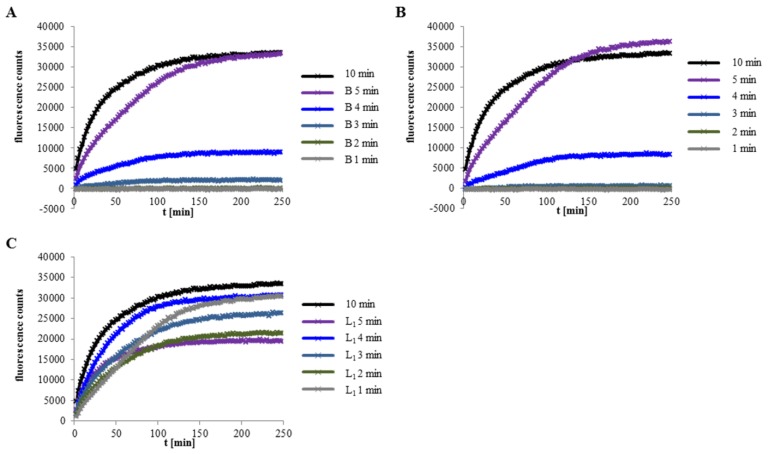
PI influx test on Vero cells. The rHbl components (37.5 pmol/mL each) were successively applied. Application order was B - L_1_ - L_2_. After B and L_1_ cells were washed twice in medium. After addition of L_2_, measurement was started immediately. (**A**) Incubation time with L_1_ was constantly 10 min, incubation times with B were decreased. (**B**) Decreasing incubation times with B as well as L_1_. (**C**) Incubation time with B was constantly 10 min, incubation times with L_1_ were decreased.

At 5, 4 and 3 min incubation time, PI influx decreased significantly, and at incubation times of less than three minutes, no more PI influx was detected (Figure 5A). A similar pattern was observed when rHbl B as well as rHbl L_1_ were applied with decreasing incubation times (Figure 5B). On the contrary, adding rHbl B constantly for 10 min and decreasing the incubation times of L_1_ led to only slightly reduced PI influx (Figure 5C). This leads to the assumption that the binding of rHbl L_1_ to B is quite rapid, as incubation times as low as 1 min were sufficient for pore formation as long as B was added for 10 min. Binding of rHbl B to the cell surface seemed to be more complex, but also possible and very efficient in 5–10 min. Due to immediate PI influx after the addition of L_2_, it is assumed that binding of this component is as rapid and efficient as the binding of L_1_ to B. These data confirm that binding of rHbl B to the target cell surface is indeed the crucial step in Hbl pore formation.

### 2.6. rHbl B Needs A Free C-Terminus for Optimum Cell Binding

**Figure 6:**
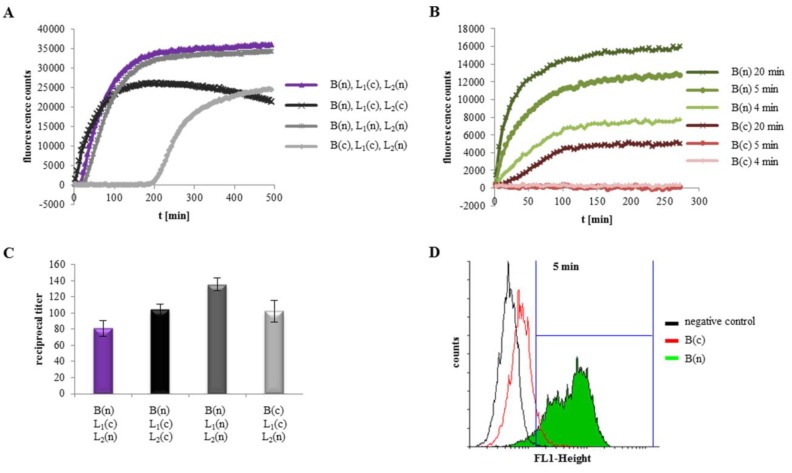
Interaction of rHbl with Vero cells. The rHbl components with N-terminal (n) as well as C-terminal (c) strep-tag were used. Proteins were applied to Vero cells in concentrations of 37.5 pmol/mL each. (**A**) Compared to the original combination—rHbl B (n), rHbl L_1_ (c) and rHbl L_2_ (n) (purple) [30]—exchange of the tag at rHbl B from the N- to the C-terminus resulted in a significantly delayed PI influx. (**B**) Consecutive application of rHbl B (n and c), L_1_ (c) and L_2_ (n); constant incubation times for L_1_ and L_2_; decreasing incubation times for the two B proteins. rHbl B (n) = green; rHbl B (c) = red. (**C**) When the different rHbl constructs were tested in WST-1 bioassays (start concentration 75 pmol/mL each, ratio 1:1:1), no reduction of toxic activity was observed. (**D**) Results of flow cytometry. rHbl B (n) = green and rHbl B (c) = red were applied to Vero cells for 5 min. mAb 1G8 directly labeled with Alexa Fluor^®^ 488 was used for detection.

To investigate the role of the N- and C-termini in Hbl activity, rHbl L_2_, L_1_ and B were used in PI influx tests, each supplied with a C-terminal or N-terminal strep-tag, respectively. Figure 6A shows that exchanging the tags on rHbl L_1_ and L_2_ did not lead to any significant differences in the velocity of pore formation compared to the initially used combination (purple). Interestingly, moving the tag on rHbl B from the N- to the C-terminus resulted in a significant delay of pore formation. That pore formation is decelerated became even more obvious when the Hbl components were applied consecutively with decreasing incubation times for rHbl B. While for rHbl B with an N-terminal tag (free C-terminus), incubation times as low as 4–5 min were sufficient for cell binding (Figure 6B; see also Figure 5A), no activity was observed for rHbl B with C-terminal tag (free N-terminus) with incubation times lower than 20 min (Figure 6B). On the other hand, no reduction of cytotoxic activity was observed in WST-1 bioassays after 24 h incubation (Figure 6C). In subsequent flow cytometry analyses, the two different rHbl B proteins were applied to Vero cells with increasing incubation times (Table 2). After only 5 min of incubation, it became obvious that rHbl B with N-terminal tag (free C-terminus) binds quicker and more effectively to the target cells than rHbl B with C-terminal tag (free N-terminus) (Table 2 and Figure 6D).

Altogether, pore formation is clearly delayed when the C-terminus of rHbl B is blocked. This is due to decelerated binding capacity to the target cell surface. Toxic activity after 24 h is not affected.

### 2.7. Hemolytic Activity of rHbl Confirms Results on Vero Cells

**Figure 7:**
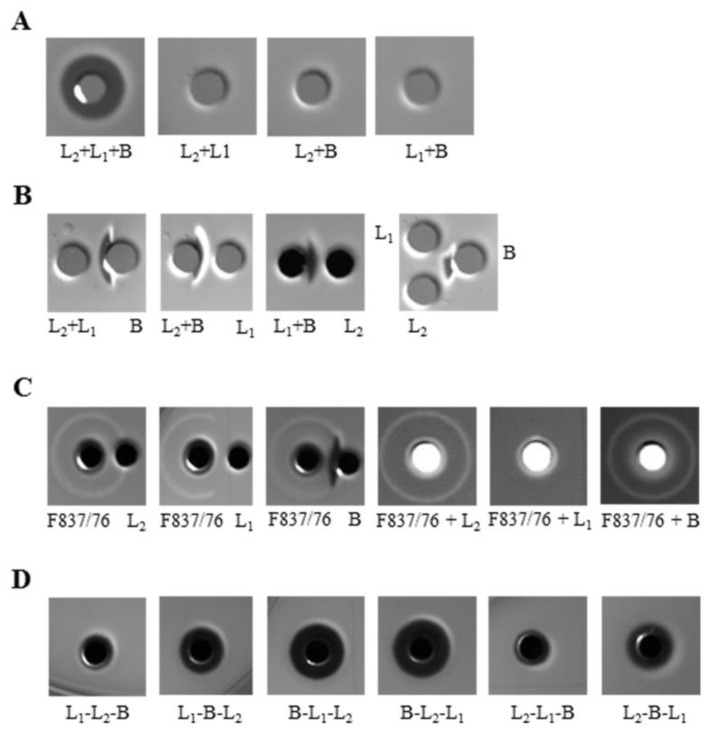
Hemolytic activity of rHbl on sheep blood agar. The rHbl stock solutions (1.5 pmol/µL) were pre-mixed, and 10 µL each were applied to 3.5 mm diameter stamp holes on sheep blood agar plates. Plates were photographed after 3–6 h of incubation at 32 °C. (**A**) All three components are necessary for hemolytic activity. (**B**) 10 µL of a single component or a mix of two were applied to 2–3 stamp holes with a distance of approximately 3 mm. (**C**) 10 µL supernatant of strain F837/76 and 10 µL of each rHbl component were applied individually to 2 stamp holes with approximately 3 mm distance or together to 1 stamp hole. (**D**) 10 µL rHbl components were successively filled into the stamp hole and incubated for 1 h each.

Analogous to previous experiments, the properties of rHbl on sheep blood agar were investigated. As expected, hemolytic activity was only detectable when all three rHbl components were present (Figure 7A). Interestingly, no discontinuous hemolysis could be observed in any experimental setup with the recombinant proteins. In the next step, the rHbl components were applied to two or three stamp holes with approximately 3 mm distance to investigate diffusion properties. Each time, the hemolysis zone was shifted towards rHbl B (Figure 7B). When rHbl was applied in 3 mm distance, or directly in the same stamp hole as the supernatant of strain F837/76, rHbl L_1_ disrupted or prevented the outer hemolysis ring, while rHbl B indeed enhanced it, and rHbl L_2_ had no detectable influence (ratios of 1:1; Figure 7C). Additionally, also sequential application of the rHbl components was tested. 10 µL of each component were successively applied to the stamp hole with an incubation time of 1 h each. The hemolysis zone was enlarged the earlier rHbl B was applied (Figure 7D). This showed that—in accordance with our earlier results—rHbl B is the key to hemolytic activity. 

## 3. Discussion

Studies on the mode of action of the three-component hemolysin BL from *B. cereus* have always been difficult. This is due to the facts that Hbl never appears in natural culture supernatants without Nhe [8,9,10], and that for a long time the generation of functional recombinant Hbl components in *E. coli* has not been possible [11]. With our recently purified rHbl proteins [30], we ultimately prove in the present study that all three Hbl components are necessary for both pore formation and cytotoxicity. One or two rHbl components did not exhibit any toxic activity, neither in WST-1 bioassays and PI influx tests nor on sheep blood agar plates. On the contrary, earlier spectrophotometric hemolysis assays detected 37% hemolysis after incubation with only Hbl L_2_ and B; and minor edema after application of only Hbl L_1_ and B in vascular permeability assays were reported [18]. 

With respect to concentration ratios, PI influx tests showed rapid pore formation with up to 10× excess of rHbl B, while depletion of B hindered pore formation. Excess of Hbl L_1_ also hindered pore formation and even hemolysis on sheep blood agar, while the amount of Hbl L_2_ seemed not to be crucial. Excess of L_2_ or L_1_ as well as depletion of B also decreased toxic activity in WST-1 bioassays (see Figure S1C). All our results point to a ratio of L_2_:L_1_:B = 1:1:10 for the fastest pore formation and 1:2:2 for maximum cytotoxicity. Contradictory to that, Sastalla and coworkers observed a loss of toxicity of supernatant of *B. cereus* strain ATCC 10876 with a decreasing amount of Hbl L_1_ at the late stationary growth phase [25]. On the other hand, already in 1991 when only two Hbl components were known, it has been shown that in relation to Hbl B, only small quantities of L are necessary to cause hemolysis [18]. Turbidity measurements confirmed that hemolysis varies with varying concentrations of Hbl B, while variations of L_2_ and L_1_ had no significant influence [29]. This coincides with the findings of our study. We also found that pore formation of Hbl into the target cell membrane is defined by the binding of the B component. 

Furthermore, we showed that pore formation of Hbl occurs within minutes. Binding of Hbl L_1_ to membrane bound Hbl B happens immediately, as we were able to reduce incubation times for rHbl L_1_ down to one minute. Also binding of Hbl L_2_ must occur instantly, as PI influx could be measured right after addition of L_2_. In earlier spectrophotometric hemolysis assays a “slow priming reaction of the B component” (3 h at 37 °C or 1 h at 42 °C) and a “rapid lytic reaction by L_1_+L_2_” (within 3 min) have been observed [24]. Our data also suggest that the priming reaction is quite fast. Depending on the protein concentration, incubation of Vero cells with rHbl B could be decreased to a minimum of 4 min, while pore formation was still detectable. Thus, we suggest that binding of Hbl B to the target cell surface is also a rapid and optimized process. Nevertheless, our WST-1 bioassays with diluted Hbl components showed that the toxicity of Hbl is defined by the B component, depending on how much B is bound to the cell surface. Thus, priming with B is the crucial step, then the lytic components L_1_ and L_2_ can easily be diluted by a factor of 10–20 without affecting the toxic activity.

In our study, only Hbl B was able to bind to the target cell surface. Thus, we found no indication for a “membrane attack complex” leading to cell lysis [24]. Furthermore we showed that neither a single component nor the combination of two components are cytotoxic, or able to form a pore. This disagrees with the idea of Madegowda and coworkers, who suggested that due to its structural homology to *E. coli* ClyA [27,28], Hbl B alone might be able to bind to the cell surface, oligomerize and form a pore, while Hbl L_2_ and L_1_ induce conformational changes, stabilize B or even enter the cell similar to anthrax toxin [26].

We showed that rHbl B is able to bind the target cell surface excellently on its own, and that the binding order is definitely B - L_1_ - L_2_. Nevertheless, rHbl B binding seemed to be enhanced in the presence of rHbl L_1_ (see Figure 4). In an earlier study we analogically observed increased binding of rHbl B in the presence of Hbl B-specific mAb 1D7, and described it as “antibody-dependent enhancement” [30,32]. Hbl L_1_ might have a similar, stabilizing effect on Hbl B binding. This observation is especially interesting considering that even before cell contact Hbl B and L_1_ form complexes in solution—the recombinant proteins as well as the natural components in *B. cereus* culture supernatants [30]. Due to additional complex formation with Hbl L_2_, we postulated that in natural *B. cereus* culture supernatants, Hbl B is mainly bound in complexes [30]. Further, free Hbl B seems to be no less important, as pore formation as well as cytotoxic and hemolytic activity were enhanced with an excess of B (this study). 

With K_D_ values of 4.7 × 10^-7^ M and 3.4 × 10^-6^ M, the Hbl L_1_-B and Hbl B-L_2_ complexes differ significantly from the one determined for NheB and C, which was 4.8 × 10^-10^ M [30,33]. Although organized in similar operons—*nheABC* and *hblC*(L_2_)*D*(L_1_)*A*(B)—and structurally related [11,34], there seem to be substantial differences in the mechanism of pore formation of the non-haemolytic enterotoxin and hemolysin BL of *B. cereus*. 

For Nhe, formation of small “pro-pores” by NheB and C (homologs to Hbl L_1_ and B) has been reported [31,33]. First, highly stable NheB-C complexes are formed in solution. Then, these complexes, as well as free C but not B, are able to bind to the target cell surface. After conformational changes, free NheB can attach. In this process, a defined level of NheBC complexes, as well as a sufficient amount of free NheB, is necessary for efficient cell binding and toxicity [31,35]. NheBC forms stable transmembrane channels with a diameter of about 2 nm, increasing membrane permeability and cytotoxic effects prior to the formation of the full pore [33]. With regards to Hbl, the corresponding B-L_1_ complexes show less stability [30], and so far, no evidence for the formation of small pores by only two components was found, although the presence of L_1_ seems to enhance the cell binding of B (this study). Furthermore, large amounts of NheC inhibit toxicity [36,37], while in the present study large amounts of the homolog Hbl B enhanced especially pore formation.

Either way, the pore is completed by attachment of the corresponding third component, NheA [31,38,39] or Hbl L_2_ [25], this study. A recent study provided evidence that NheA is highly flexible and undergoes major conformational changes during pore formation [38]. The mechanism of the Hbl L_2_ attachment to B and L_1_ is yet to be determined. For Nhe, it has been shown that maximum toxicity is reached at a concentration ratio of NheA:B:C = 10:10:1, but with strain-specific differences [36,37,38]. On the contrary, in the present study a molar ratio of Hbl L_2_:L_1_:B = 10:10:1 did not result in any toxic or pore-forming activity of Hbl. 

According to the current knowledge, the mechanism of Hbl is modeled as follows: First, Hbl B binds to the target cell surface. This is the crucial and most complex step in Hbl pore formation, but is still completed within a few minutes. In natural culture supernatants of *B. cereus*, large amounts of Hbl B and L_1_ are present as complexes [30]. Linked with L_1_, the binding capacity of B to the target cell surface is increased. In a subsequent step, free Hbl L_1_ binds to B. This is a very efficient and quick process, as well as the adjacent binding of Hbl L_2_. Strong excess of Hbl L_1_ hinders pore formation. On the other hand, it is still unclear how many molecules of each component are necessary for pore formation, if conformational changes appear, and which protein moieties are involved in the interaction.

## 4. Materials and Methods 

### 4.1. Bacterial Strains and Culture Conditions

The *B. cereus* strain F837/76 (DSM 4222) was used in this study. To obtain toxin-rich culture supernatant, the strain was grown in CGY medium with 1% glucose for 6 h at 32 °C with shaking. According to [40], the sample was centrifuged (4.000× g at 4 °C for 20 min) before addition of 1 mM EDTA and filtration through a 0.2 µm sterile filter. *E. coli* strain BL21 (DE3) was used for overexpression of recombinant (r) Hbl proteins.

### 4.2. Production of Purified, Recombinant (r) Hbl Components

Hbl proteins were overexpressed in the *E. coli* strain BL21 (DE3) and purified as described earlier, resulting in rHbl B and L_2_ with N-terminal strep-tag as well as rHbl L_1_ with C-terminal strep-tag [30]. In this study, the tags were furthermore exchanged to the respective other terminus. For that, *hblC* (L_2_), *hblD* (L_1_) and *hblA* (B) were amplified by PCR using pASK-IBA5+hblC, pASK-IBA3+hblD and pASK-IBA5+hblA as templates, as well as the primer pairs HblL_2_-fw-SacII (ATA TCC GCG GAT GCA AGC AGA AAC TCA ACA AGA AAA C) and HblL_2_-rev-NcoI-GC (ATA TCC ATG GGC AAA TTT ATA CAC TTG TTC TTC AAG G), HblL_1_-fw-KpnI (ATA TGG TAC CCG CAC AAG AAA CGA CCG CTC A) and HblL_1_-rev-NcoI (ATA TCC ATG GCT ACT CCT GTT TAA AAG CAA TAT CTT), and HblB-fw-SacII (ATA TCC GCG GAT GGC AAG TGA AAT TGA ACA AAC G) and HblB-rev-NcoI-GC (ATA TCC ATG GGC TTT TTG TGG AGT AAC AGT TTC TAC). The genes lacking the sequences for the N-terminal signal peptides for secretion [41,42] were cloned into pASK-IBA5+ or pASK-IBA3+ (iba lifesciences, Göttingen, Germany), respectively. The constructs were sequenced using the primers pASK-IBA-seq-fw (CACTCCCTATCAGTGATAG) and pASK-IBA-seq-rev (GCACAATGTGCGCCAT).

As rHbl L_2_ with C-terminal strep-tag could not be purified via affinity chromatography, its concentration was estimated via enzyme immuno assay (see [43,44]) and the crude *E. coli* cell extract was used. The cell extract applied solely did not lead to any pore formation or toxic activity (data not shown).

### 4.3. Cell Line and Culture Conditions

Vero cells were obtained from ECACC (European Collection of Cell Cultures). They were cultured in 80 cm^2^ culture flasks in a humidified incubator at 37 °C and 7% CO_2_ in MEM Earle’s medium (Biochrom GmbH, Berlin, Germany) plus supplements, as recommended by the supplier.

### 4.4. Cytotoxicity Assays

Cytotoxicity assays were performed as previously described [40,43,45]. Briefly, for WST-1 bioassays serial dilutions of *B. cereus* culture supernatant or rHbl proteins were placed into 96 well plates (0.1 mL per well) and subsequently, Vero cell suspensions (1 × 10^4^ cells and 0.1 mL per well) were added. After 24 h incubation at 37 °C and 7% CO_2_, cell viability was determined by the addition of WST-1 (Roche diagnostics, Penzberg, Germany). Optical density was recorded in a Tecan photometer at 450 nm using Ridawin software. Dose-response curves were calculated to obtain 50% lethal concentrations, which are shown as reciprocal titers. 

To assess pore formation in the membranes of Vero cells, propidium iodide (PI) influx tests were used. 4 × 10^4^ Vero cells were seeded in 200 µL MEM Earle’s medium /well in black 96 well plates and incubated for 24 h at 37 °C and 7% CO_2_. 100 µL medium were removed and 100 µL fresh medium were added containing 10 µg/mL PI (Sigma-Aldrich Biochemie GmbH, Hamburg, Germany) and *B. cereus* culture supernatant (1:20) or rHbl components in appropriate dilutions. Fluorescence was measured immediately in a Victor 1420 multilabel counter (Perkin Elmer, Boston, MA, USA) for 4 h every 2.5 min (excitation: 530 nm; emission: 616 nm; excitation time: 1s; excitation strength: 20,000). Fluorescence curves of three replicates were calculated using Microsoft Excel. The start point of PI incorporation and the slope of the fluorescence curves allow concluding on the efficiency and velocity of pore formation (see also [36,40]), while differences in the end point fluorescence rather depend on the target cells. 

Stock solutions of the rHbl components were pre-mixed in appropriate ratios and added as serial dilutions (WST-1 bioassay; start concentrations as indicated in the respective experiments) or in concentrations of 37.5 pmol/mL each (PI) to the cells.

### 4.5. Flow Cytometry Analyses

For flow cytometry analyses, Vero cells were counted and adjusted to 1 × 10^6^ cells in 500 µL EC buffer (140 mM NaCl, 15 mM HEPES, 1 mM MgCl_2_, 1 mM CaCl_2_, 10 mM glucose, pH 7.2). rHbl B ± rHbl L_1_ or L_2_ were added in EC buffer in concentrations of 9.38 pmol/mL each to a total volume of 1 mL. After 1 h incubation at 37 °C with moderate agitation, 2 mL 1% BSA-PBS were added and cells were centrifuged for 5 min at 800 rpm. Cells were washed again in 2 mL 1% BSA-PBS. Samples were incubated with 5 µg/mL mAb 1G8 or 1B8 [30,43] in 1 mL 1% BSA-PBS for 1 h at RT and again washed twice with 2 mL 1% BSA-PBS. Subsequently, samples were incubated with 2 µg/mL Alexa Fluor^®^ 488 goat anti mouse IgG (life technologies, Carlsbad, CA, USA) for 45 min at 4 °C. Alternatively, mAb 1G8 directly labeled with Alexa Fluor^®^ 488 was used for detection. After two washing steps with 2 mL 1% BSA-PSB, cells were resuspended in 500 µL 1% BSA-PBS, and transferred to flow cytometry tubes. For the detection of Hbl L_2_, Vero cells were first incubated with rHbl B, L_1_ or B+L_1_ (4.17 pmol/mL each) and in a second step with a mixture of rHbl L_2_ (4.17 pmol/mL) and the neutralizing mAb 1H9 (5 µg/mL) [30]. Fluorescence was measured in a FACS Calibur using the CellQuestPro software (BD Bioscience, San Jose, CA, USA). Cell populations were visualized in the FSC SSC dot-plot. Fluorescence was recorded in fluorescence channel 1 and shown as a histogram.

### 4.6. Determination of Hemolytic Activity

10 µL of toxin containing F837/76 supernatant or rHbl L_2_, L_1_ and B (1.5 pmol/µL each) were filled individually or as mixture in different ratios into 3.5 mm diameter stamp holes on sheep blood agar plates. After 3–6 h incubation at 32 °C hemolysis patterns were assessed and documented.

## Figures and Tables

**Table 1:** Results of flow cytometry demonstrating binding of rHbl B to Vero cells. Cells were incubated with the respective rHbl components, mAbs 1G8, 1B8 or 1H9 and Alexa Fluor^®^ 488 goat anti mouse IgG. FL1: fluorescence at 488 nm.

Sample	FL1 Positive (%)
mAb 1G8 (anti Hbl B)	8.26 ± 0.71
rHbl B	2.05 ± 0.28
rHbl B + 1G8	97.46 ± 0.59
mAb 1B8 (anti Hbl B)	2.03±0.05
rHbl B + 1B8	39.93±1.97
rHbl B + L_1_ + 1B8	82.31±3.25
rHbl B + L_2_ + 1B8	29.62±0.78
mAb 1H9 (anti Hbl L_2_)	1.25 ± 0.35
L_2_+1H9	3.5 ± 0.35
B + L_2_+1H9	3.02 ± 0.06
L_1_ + L_2_+1H9	3.33 ± 0.09
B+L_1_ + L_2_+1H9	93.66 ± 0.55

**Table 2:** Flow cytometry on Vero cells shows different cell binding of rHbl B with free C- or N-terminus. Cells were incubated with rHbl B with an N-terminal (n) or C-terminal (c) strep-tag, respectively, for different times, and subsequently with mAb 1G8 directly labeled with Alexa Fluor^®^ 488. FL1: fluorescence at 488 nm.

Sample		FL1 Positive (%)
mAb 1G8 (anti Hbl B)		0.48 ± 0.15
rHbl B (n)	5 min	93.38 ± 1.38
	15 min	93.44 ± 2.29
	30 min	93.04 ± 4.1
rHbl B (c)	5 min	8.92 ± 2.36
	15 min	100 ± 5.94
	30 min	100 ± 7.05

## Supplementary Materials

The following are available online at https://www.mdpi.com/2072-6651/11/5/281/s1, Figure S1: Additional results on cytotoxicity and cell binding of *B. cereus* Hbl.
