# Supplementary material for: Binding to The Target Cell Surface Is The Crucial Step in Pore Formation of Hemolysin BL from Bacillus cereus
Source: Toxins (Basel). 2019 May 20;11(5):281. doi: 10.3390/toxins11050281 (PMC6563250; doi:10.3390/toxins11050281)
Supplement: Supplementary file 1 [file toxins-11-00281-s001.pdf]

Supplementary Materials: Binding to The Target Cell Surface is The Crucial Step in Pore Formation of Hemolysin BL from *Bacillus cereus*

Nadja Jessberger, Richard Dietrich, Stefanie Schwemmer, Franziska Tausch, Valerie Schwenk, Andrea Didier and Erwin Märtlbauer

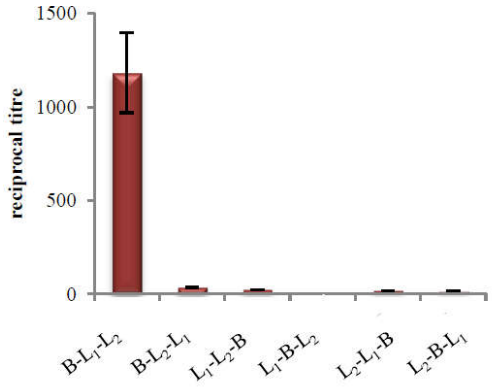

(**A**)

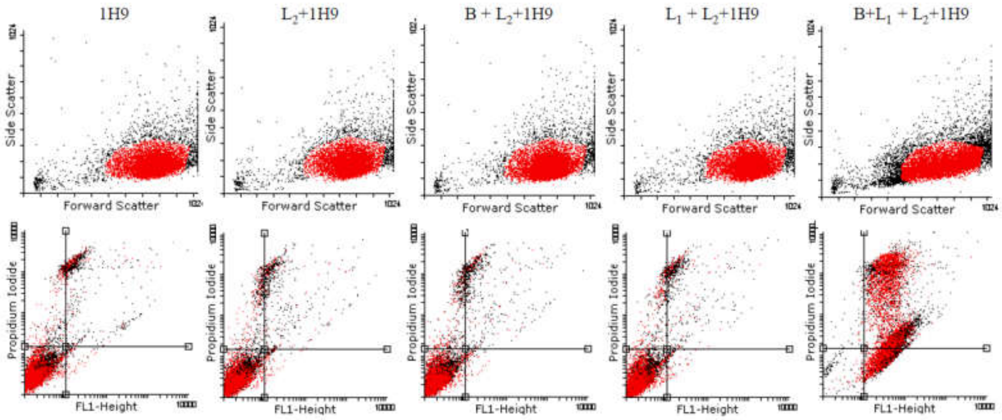

PI positive (%):14.11 ± 3.24 10.85 ± 1.77 10.8 ± 0.41 9.83 ± 0.18 71.31 ± 6.93

(**B**)

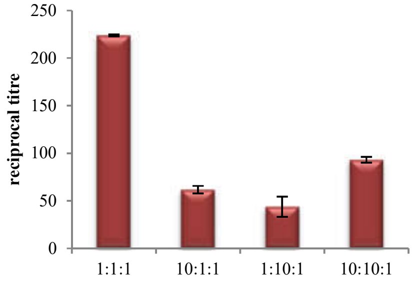

(**C**)

Figure S1. Additional results on cytotoxicity and cell binding of *B. cereus* Hbl. (**A**) WST-1 bioassay. rHbl components were applied to Vero cells consecutively with two intermediate washing steps. rHbl B and L_1 were applied constantly (3.75 pmol/mL), L_2 as serial dilution, each for 1 h. After the third component, cells were again washed two times and incubated with WST-1 for 1.5 h. titers were determined as the toxin dilution causing 50% dead cells. (**B**) Flow cytometry on Vero cells. Cells were incubated with different combinations of rHbl B, L_1, L_2, and mAb 1H9 as well as 5 µg/mL PI and Alexa Fluor® 488 goat anti mouse IgG for detection. Upper row: cell size and granularity. Lower

row: FL1 (488 nm, see Table 1) and propidium iodide (PI) signals. (**C**) rHbl components were applied in different concentration ratios as dilution series to Vero cells in WST-1 bioassays. 1:1:1 = 75 pmol/mL each as start dilution. 10:1:1 = 10× excess of rHbl L_2. 1:10:1 = 10× excess of rHbl L_1. 10:10:1 = 10× depletion of rHbl B.
